# Concurrent paclitaxel/cisplatin chemoradiotherapy with or without consolidation chemotherapy in high-risk early-stage cervical cancer patients following radical hysterectomy: preliminary results of a phase III randomized study

**DOI:** 10.18632/oncotarget.10450

**Published:** 2016-07-06

**Authors:** Hongqin Zhao, Lili Li, Huafang Su, Baochai Lin, Xuebang Zhang, Shengliu Xue, Zhenghua Fei, Lihao Zhao, Qintuo Pan, Xiance Jin, Congying Xie

**Affiliations:** ^1^ Departments of Gynecology, The First Affiliated Hospital of Wenzhou Medical University, Wenzhou, China, 325000; ^2^ Departments of Radiation Oncology and Chemotherapy, The First Affiliated Hospital of Wenzhou Medical University, Wenzhou, China, 325000; ^3^ Departments of Eye Fundus Surgery, Eye Hospital of Wenzhou Medical University, Wenzhou, China, 325002

**Keywords:** cervical cancer, chemoradiotherapy, consolidation chemotherapy, cisplatin, paclitaxel

## Abstract

A phase III randomized study on the efficacy and safety of consolidation chemotherapy with paclitaxel plus cisplatin following radical hysterectomy and adjuvant chemoradiotherapy (CRT) in the treatment of high risk early-stage cervical cancer were reported. 146 eligible patients were randomized to arm A receiving concurrent CRT or arm B receiving CRT plus consolidation chemotherapy, respectively. An interim analysis showed a trend of improvement on disease-free survival (DFS) and overall survival (OS) in arm B with hazard ratios (HR) of 1.25 (95% CI = 0.60–2.60, *p* = 0.55) and 1.43 (95% CI = 0.64–3.20, *p* = 0.38) for DFS and OS, respectively. The 3-year DFS and OS were 82.0% vs.74.3%, and 86.6% vs. 78.3% for patients receiving CRT plus consolidation chemotherapy and CRT alone, respectively. There was significant difference between the two arms in distant alone recurrence (*p* = 0.048). Multivariate analysis indicated that pathologic type was a significant prognostic factor for OS (*p* = 0.045), positive pelvic nodes were significantly associated with both OS (*p*=0.02) and DFS (*P*=0.03). Grade 2 to 4 gastrointestinal disorder (*p* = 0.95), radiation enteritis (*P*=0.48), radiation cystitis (*p* = 0.27) and radioepidermitis (*p* = 0.46) were similar in the two arms. Overall rates of grade 0–2/3–4 myelosuppression were 87.7%/12.3% for arm A and 74.6%/25.4% for arm B, respectively, but this difference was not statistically significant (*p* = 0.05). In conclusion, concurrent CRT plus consolidation chemotherapy may play a potential role in further improving survival outcomes for high-risk early stage cervical cancer patients compared CRT alone.

## INTRODUCTION

Cervical cancer is one of the most common gynecologic cancers worldwide. Approximately 83% of the cases happened in the developing countries [1]. Early stage cervical cancer can be effectively treated with radiotherapy or radical hysterectomy plus pelvic lymph node dissection. However, several pathological risk factors, such as lymph node metastasis, positive vaginal resection margin and parametrial invasion have been identified as high risk factors that will compromise patients' prognosis [2, 3]. Besides, large tumor diameter, deep stromal invasion and lymphovascular space involvement are also considered as intermediate risk factors of recurrence [4–6].

According to the National Comprehensive Cancer Network (NCCN) guidelines, concurrent radiotherapy with cisplatin-based chemotherapy has become the standard treatment for early stage cervical cancer patients with positive pelvic nodes and/or positive surgical margin and/or positive parametrium [7, 8]. Concurrent chemoradiotherapy (CRT) decreased both the rate of local and distant failure since chemotherapy can act as a radiation sensitizer [9]. Although patients with early cervical cancer achieved relatively high survival rates, many patients with pathological risk factors treated with CRT still suffered from local or distant relapse. How to improve the treatment outcome of these patients is a major concern and requires further clinical investigation.

Consolidation chemotherapy after the standard CRT treatment is aimed to eradicate residual disease, including occult disease outside the pelvic radiation field. Several clinical trials have been conducted to explore the potential roles of consolidation chemotherapy in the treatment of cervical cancer [10–12]. A 19% absolute improvement on 5-year OS had been reported in trials with additional chemotherapy following CRT [13]. Paclitaxel has been demonstrated to be a good radiosensitizer. Paclitaxel/cisplatin combination chemotherapy was demonstrated to have superior progression-free survival than platinum alone in some phase III studies [14]. Therefore, we performed a phase III randomized trial to investigate the efficacy and safety of postoperative concurrent CRT with paclitaxel/cisplatin plus additional consolidation chemotherapy in the treatment of high-risk early-stage cervical cancer patients following radical hysterectomy.

## RESULTS

### Patients' characteristics

**Figure 1:**
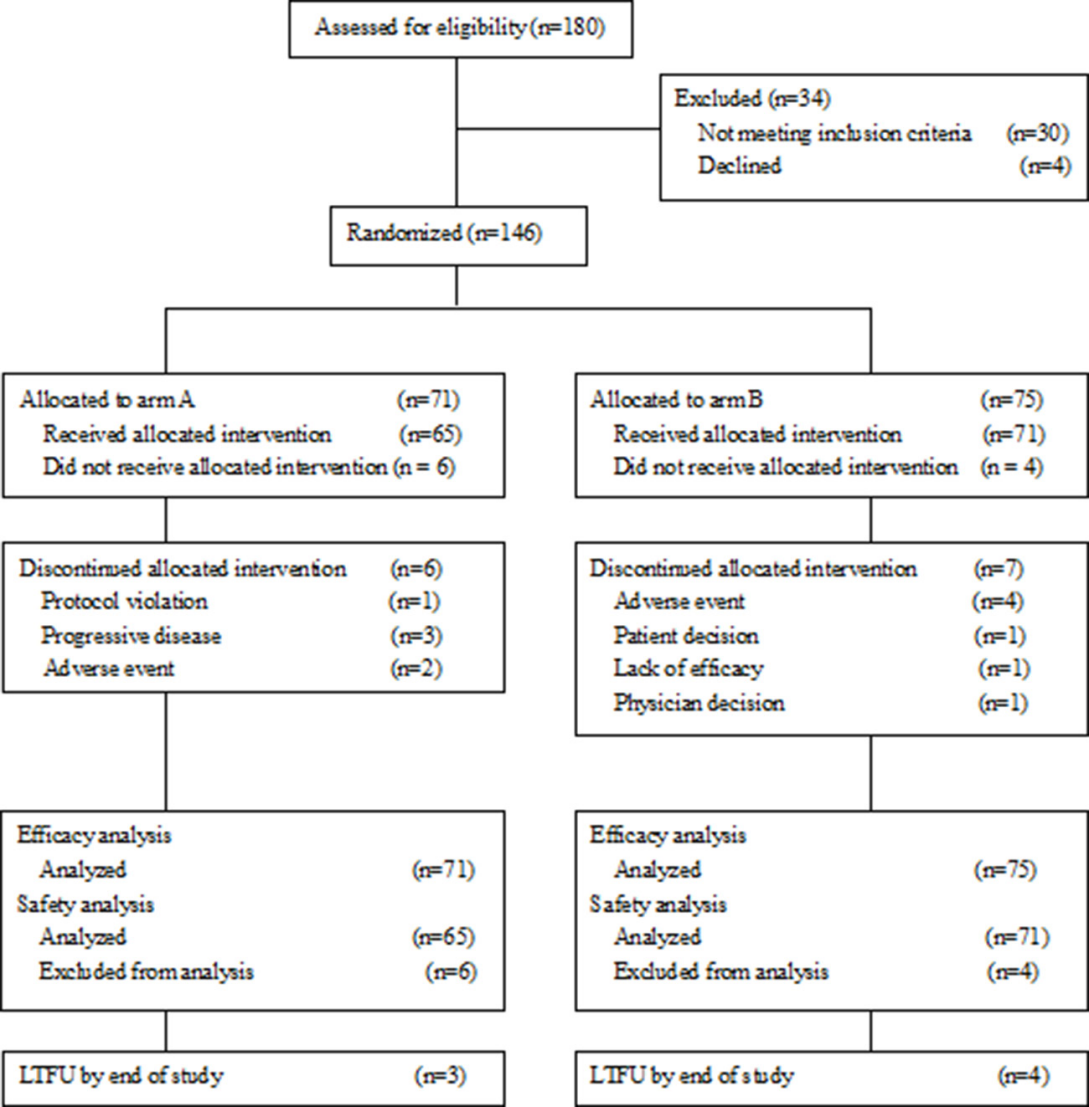
CONSORT diagram of the study design Arm A treatment consisted of paclitaxel plus cisplatin chemotherapy combined with radiotherapy. Arm B treatment consisted of paclitaxel plus cisplatin chemoradiotherapy followed by paclitaxel plus cisplatin consolidation chemotherapy. LTFU: lost to follow-up.

From January 2011 to November 2014, 146 women with an age from 28–75 years old were enrolled and randomly assigned to arm A (*n* = 71) or arm B (*n* = 75). These patients composed the intent-to-treat population. Patients in arm A treated with CRT only. Patients in arm B treated with CRT plus consolidation chemotherapy. Ten eligible patients who did not receive allocated intervention were excluded from the clinical trial (six in arm A and four in arm B). Five patients withdrew their consents. Three patients moved away and lost follow-up, two died from other causes. There were 65 patients in arm A and 71 patients in arm B included in the safety analysis. The follow-up phase lasted until July 2015 with a median follow-up time of 30 months. The study CONSORT diagram is presented in Figure 1. Clinical characteristics were well balanced at baseline between treatment arms and presented in Table 1.

**Table 1:** Demographics and clinical characteristics of enrolled patients

Characteristics	All patients		Arm A		Arm B		*p*-value
	*N* = 146	%	*N* = 71	%	*N* = 75	%	
Age							
Median	51		52		50		0.78
Range	28–75		30–73		28–75		
Histology							
SCC	106	72.6	51	71.8	55	73.3	0.84
Non-SCC	40	27.4	20	28.2	20	26.7	
FIGO stage							
IA2	16	10.9	6	8.5	10	13.1	0.61
IB	69	47.2	36	50.7	33	44	
IIA	43	29.6	19	26.8	24	32	
MIIB	18	12.3	10	14	8	10.7	
Differentiation							
Well	14	9.6	4	5.6	10	13.3	0.21
Moderate	91	62.3	44	62	47	62.7	
Poor	41	28.1	23	32.4	18	24	
Largest diameter							
Mean	2.9		2.8		3		0.54
Range	0.5–6.0		0.5–5.8		0.5–6.0		
Number of positive pelvic nodes							
0	27	18.5	16	22.5	11	14.7	0.29
1–2	76	52.1	36	50.7	40	53.3	
3–4	24	16.4	13	18.3	11	14.7	
> 4	19	13	6	8.5	13	17.3	
Lymphovascular invasion							
Negative	74	50.7	39	54.9	35	46.7	0.32
Positive	72	49.3	32	45.1	40	53.3	
Stromal invasion depth							
< 1/3	28	19.2	16	22.5	12	16	0.32
> 1/3	118	80.8	55	77.5	63	84	
Positive parametrium							
Yes	17	11.6	9	12.7	8	10.7	0.71
No	129	88.4	61	87.3	67	89.3	
Positive surgical margin							
Yes	6	4.1	4	5.6	2	2.7	0.37
No	140	95.9	67	94.3	73	97.3	

Abbreviation: FIGO, International Federation of Gynecology and Obstetrics; SCC, squamous cell carcinoma; Non-SCC group consists of 20 adenosquamous cell carcinomas, 16 adenocarcinomas, and 4 mixed type.

### Efficacy and prognostic factors

Table 2 lists the administered chemotherapy and radiotherapy. The average number of cycles of cisplatin and paclitaxel in arm A and arm B were two (range, one to two doses) and three (range, two to four), respectively. In arm A, 59 patients (90.8%) completed the chemotherapy during CRT, while 64 patients (90.1%) in arm B finished allocated chemotherapy in the consolidation chemotherapy phase. One patient received 5-FU instead of paclitaxel because of drug allergy. The median external radiotherapy dose was 48Gy (46–50 Gy) in both arms. Eight patients with documented common iliac lymph nodes involvement received extended field external-beam radiotherapy (EBRT), two in arm A and six in arm B, respectively.

**Table 2:** Details of chemotherapy and radiation treatment delivered

Treatment	Arm A^a^ (*n* = 65)	Arm B^b^ (*n* = 71)	All patients (*n* = 136)
Chemoradiotherapy phase			
Number of patients	59	66	125
Percentage (%)	90.8	93	91.9
Cycle numbers of paclitaxel/cisplatin			
Range	1–2	1–2	1–2
Consolidation chemotherapy			
Number of patients	-	64	64
Percentage (%)	-	90.1	47.1
Cycle numbers of paclitaxel/cisplatin for consolidation chemotherapy			
Range	-	2–4	-
Radiation therapy			
Number of patients	65	71	136
Percentage (%)	100	100	100
Duration of external beam radiotherapy (days)			
Median	39	38	39
Range	35–45	33–46	35–46
Total external beam radiotherapy dose (Gy)			
Median	48	48	48
Range	46–50	46–50	46–50

a Arm A consisted of paclitaxel plus cisplatin chemotherapy combined with radiotherapy.

b Arm B consisted of paclitaxel plus cisplatin chemoradiotherapy followed by paclitaxel plus cisplatin consolidation chemotherapy.

At the time of this interim analysis, 29 (21.3%) patients experienced treatment failure, in which 12 had locoregional failures, 12 had distant relapse alone and 5 had both. Patterns of failure were summarized in Table 3 with a median recurrence time of 25.5 months (range, 3–61 months). There were 25 deaths reported until our last follow up. In arm A, metastases were found in the pelvic (*n* = 5), lung (*n* = 8) and bone (*n* = 1). One patient had both pelvic recurrence and lung metastasis. One patient had both pelvic recurrence and bone metastasis. In arm B, metastases were found in the pelvic (*n* = 7), lung (*n* = 1), bone (*n* = 1) and supraclavicular lymph nodes (*n* = 1). One patient had both pelvic recurrence and lung metastasis, one had pelvic recurrence and liver metastasis, and one had both pelvic recurrence and upper abdominal metastasis. There was significant difference between the two arms in distant alone recurrence (*p* = 0.048).

**Table 3:** Failure patterns

Failure pattern	Arm A^a^ (*n* = 65)		Arm B^b^ (*n* = 71)		*p*
	No.	Percentage(%)	No.	Percentage(%)	
Locoregional	5	9.2	7	9.9	0.66
Para-aortic region alone	0	0	0	0	-
Distant alone^c^	9	13.8	3	2.8	0.048
Locoregional and distant^d^	2	1.5	3	5.6	0.72
Total relapse	16	24.5	13	18.3	0.37

a Arm A consisted of paclitaxel plus cisplatin chemotherapy combined with radiotherapy.

b Arm B consisted of paclitaxel plus cisplatin chemoradiotherapy followed by paclitaxel plus cisplatin consolidation chemotherapy.

c Site(s) of metastasis other than para-aortic lymph nodes or para-aortic node metastasis plus other distant site(s).

d Locoregional recurrence plus any extrapelvic metastasis including para-aortic lymph nodes.

**Figure 2:**
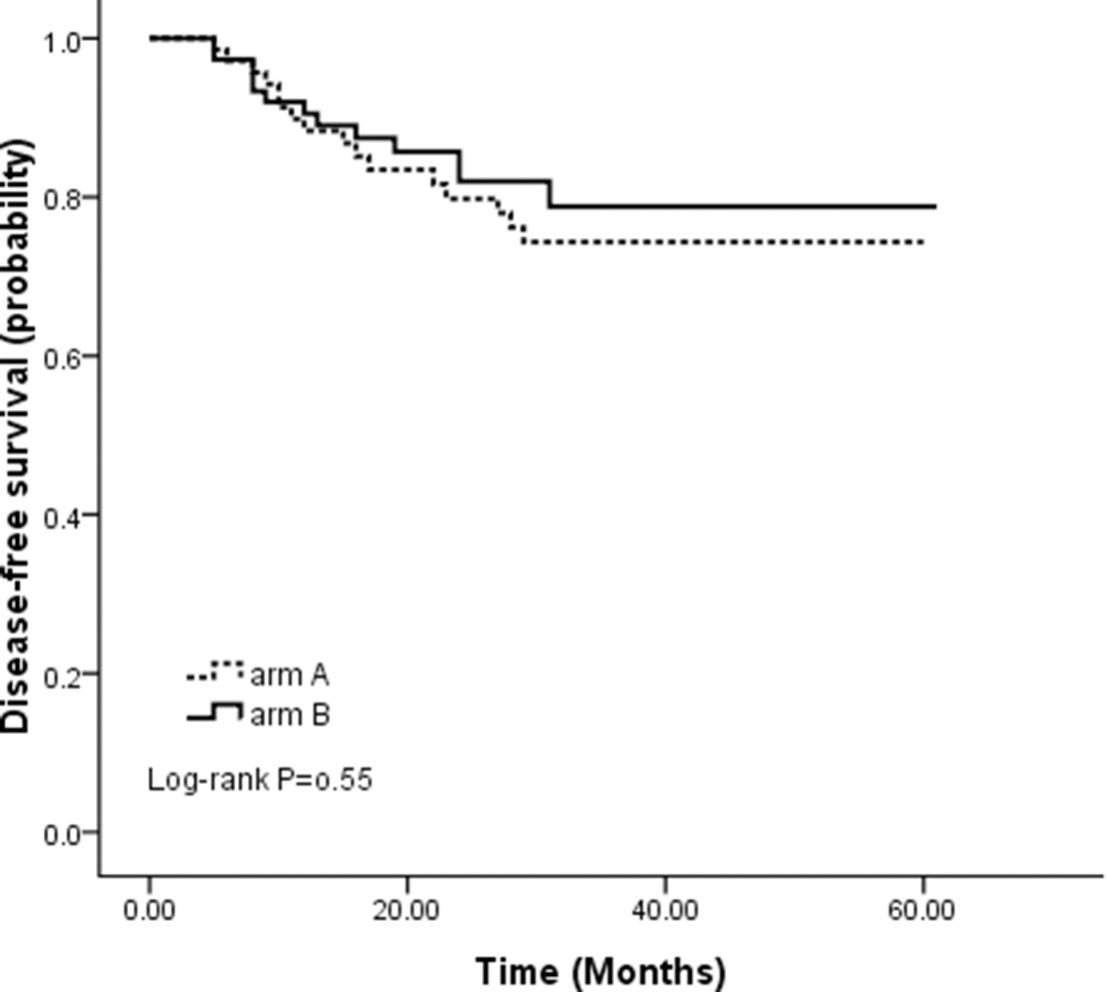
Disease-free survival for 71 patients randomized to arm A and for 75 patients randomized to arm B

**Figure 3:**
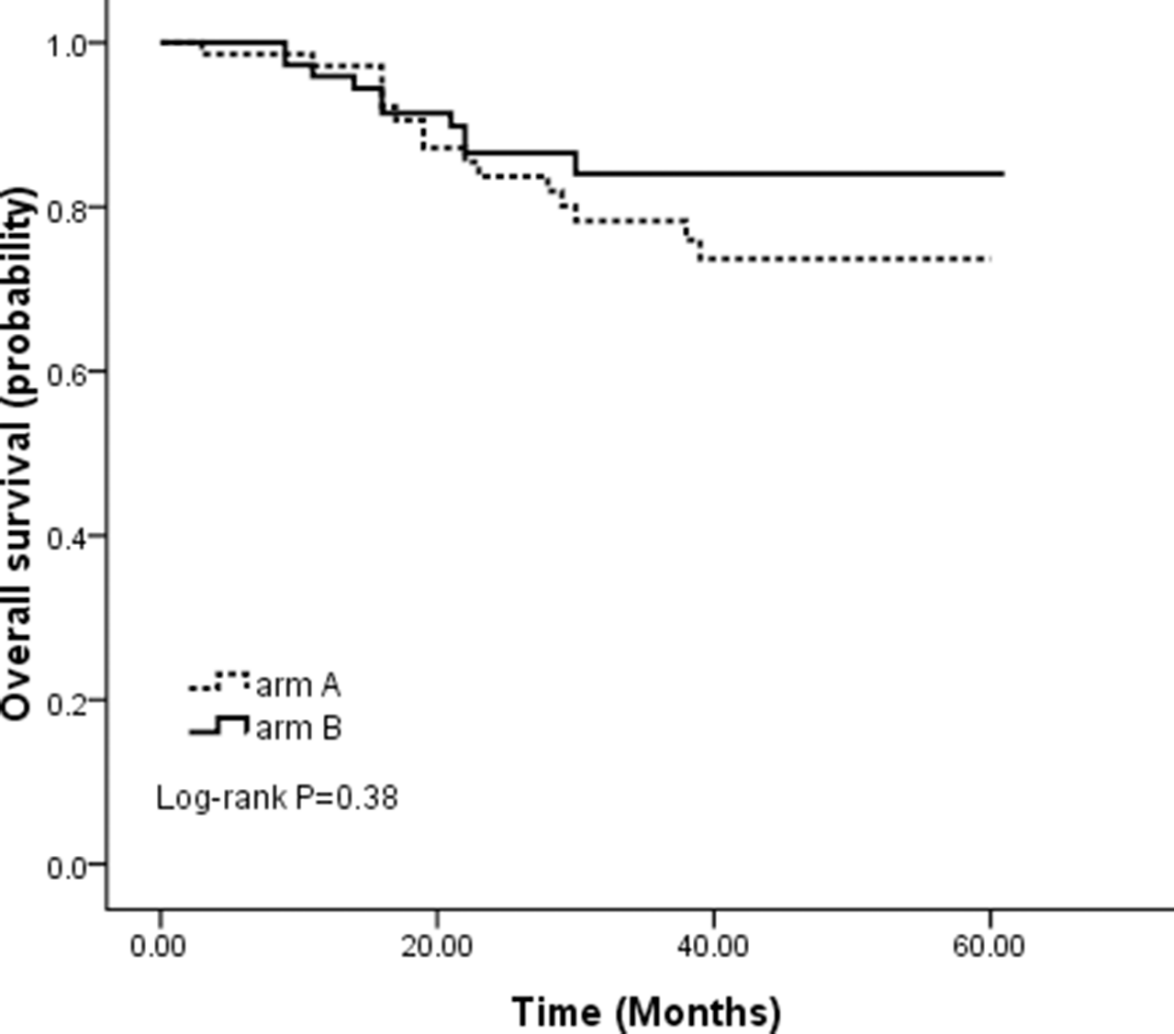
Overall survival for 71 patients randomized to arm A treatment and for 75 patients randomized to arm B

Figure 2 presents the Kaplan-Meier depiction of DFS by treatment arms. Patients in arm B had an insignificant improvement in DFS compared with those in arm A with an estimated 3-year DFS of 82.0% and 74.3% for arm B and arm A, respectively. Figure 3 shows the OS by treatment groups. Similarly, there was no significant difference between the two arms with an estimated 3-year OS of 86.6% and 78.3% for arm B and arm A, respectively. The hazard ratios of Cox model analysis projected arm A versus arm B were 1.25 (95% CI = 0.60–2.60, *p* = 0.55) and 1.43 (95% CI = 0.64–3.20, *p* = 0.38) for DFS and OS, respectively.

Univariate analysis indicated pathologic type and positive pelvic nodes were significantly associated with both DFS and OS (Table 4). Age, tumor size, positive parametrium, positive surgical margin, stromal invasion depth, lymphovascular invasion and treatment did not show statistically significant differences for either OS or DFS. In multivariate analysis, pathologic type and positive pelvic nodes were shown to be prognostic factors significantly associated with OS (*p* = 0.045 and *p* = 0.02, respectively). DFS was also affected by positive pelvic nodes (*P* = 0.03).

**Table 4:** Prognostic analysis of disease-free survival (DFS) and overall survival (OS)

Variables	Subgroup	DFS			OS		
		Univariate	Multivariate		Univariate	Multivariate	
		*p*	HR (95% CI)	*p*	*p*	HR (95% CI)	*p*
Age (yr)	< 51 vs. ≥ 51	0.15	-	-	0.86	-	-
Tumor size (cm)	< 3 vs. ≥ 3	0.08	2.41 (0.80–6.67)	0.265	0.06	1.77 (0.56–9.43)	0.51
Pathologic type	SCC vs. non-SCC	0.04	2.29 (0.67–10.31)	0.13	0.04	3.31 (1.15–7.60)	0.045
Positive pelvic nodes	No vs. yes	0.04	3.7 (1.21–9.63)	0.031	0.03	2.96 (1.78–8.64)	0.02
Positive parametrium	No vs. yes	0.82	-	-	0.70	-	-
Positive surgical margin	No vs. yes	0.39	-	-	0.41	-	-
Stromal invasion depth	< 1/3 vs. > 1/3	0.58	-	-	0.43	-	-
Lymphovascular invasion	No vs. yes	0.07	2.30 (0.90–7.85)	0.182	0.14	-	-
Treatment	Arm A vs. arm B	0.55	-	-	0.38	-	-

Abbreviation: DFS, disease-free survival; OS, overall survival; HR, hazard ratio; CI, confidence interval; SCC, squamous cell carcinoma,

a Arm A consisted of paclitaxel plus cisplatin chemotherapy combined with radiotherapy.

b Arm B consisted of paclitaxel plus cisplatin chemoradiotherapy followed by paclitaxel plus cisplatin consolidation chemotherapy.

### Toxicities

The incidences and categories of adverse events were listed in Table 5. Generally, grade 3 and 4 toxicities, including myelosuppression and gastrointestinal disorder were infrequent. Grade 2 to 4 gastrointestinal disorders (*p* = 0.95), radiation enteritis (*P* = 0.48), radiation cystitis (*p* = 0.27) and radioepidermitis (*p* = 0.46) were similar in the two arms. Overall rates of grade 0–2/3–4 myelosuppression were 87.7%/12.3% for arm A and 74.6%/25.4% for arm B, respectively, but this difference was not statistically significant (*p* = 0.05). Late toxicities were rare and manageable overall.

**Table 5:** Toxicities comparison between Arm A (standard chemoradiotherapy) and Arm B (standard chemoradiotherapy plus consolidation chemotherapy)

Toxicity/number of patients (%)	Arm A^a^ (*N* = 65)										Arm B^b^ (*n* = 71)									
	Grade 0		Grade 1		Grade 2		Grade 3		Grade 4		Grade 0		Grade 1		Grade 2		Grade 3		Grade 4	
	No.	%	No.	%	No.	%	No.	%	No.	%	No.	%	No.	%	No.	%	No.	%	No.	%
Gastrointestinal reaction	8	12.3	10	15.4	41	63.1	2	3.1	4	6.1	0	0	20	28.2	43	60.6	2	2.8	6	8.4
Radiation enteritis	39	60	16	24.6	10	15.4	0	0	0	0	20	28.2	43	60.6	8	11.2	0	0	0	0
Radiation cystitis	45	69.2	4	6.2	16	24.6	0	0	0	0	39	54.9	20	28.2	12	16.9	0	0	0	0
Radioepidermitis	30	46.2	27	41.5	8	12.3	0	0	0	0	24	33.8	41	57.7	6	8.5	0	0	0	0
Myelosuppression	12	18.5	19	29.2	26	40	8	12.3	0	0	1	1.4	18	25.4	34	47.9	18	25.3	0	0

a Arm A consisted of paclitaxel plus cisplatin chemotherapy combined with radiotherapy.

b Arm B consisted of paclitaxel plus cisplatin chemoradiotherapy followed by paclitaxel plus cisplatin consolidation chemotherapy.

## DISCUSSION

Since 1999, randomized trials have reported that patients with cervical cancer treated by concurrent CRT had a significant survival advantage compared with those treated by radiotherapy alone [15]. However, cervical cancer patients with high risk factors still suffer from 20–30% chance of local failure [16, 17] and 18–25% of distant failure [17, 18] after CRT. RTOG 90–01 demonstrated that CRT could decrease the para-aortic recurrence with a long-term follow-up, however, more than 50% of patients were found to have distant metastasis [18]. Therefore, additional researches focused on the role of consolidation chemotherapy after standard concurrent CRT are necessary. In this study, the efficacy and safety of concurrent Paclitaxel/Cisplatin chemoradiotherapy with or without consolidation chemotherapy in high-risk early-stage cervical cancer patients after radical hysterectomy were investigated in a phase III randomized trial. An improvement on DFS and OS in patients treated by concurrent paclitaxel/cisplatin CRT with consolidation chemotherapy were demonstrated compared with those treated by CRT alone, although no statistical significance was observed.

A statistically significant survival advantage in patients with advanced cervical cancer treated by adding weekly concurrent gemcitabine and 2 cycles of adjuvant gemcitabine and cisplatin to standard CRT compared with standard single agent CRT alone (OS: HR 0.68, 95% CI 0.49 to 0.95, *p* = 0.02) was demonstrated in Dueñas-González's trial [10]. The local failure was not improved in the study arm (11.2% vs. 16.4%, *p* = 0.10), while the difference in the distant failure was significant (8.1% vs. 16.4%, *p* = 0.005). Similarly, in the present study, the OS (HR 1.43; 95% CI = 0.64–3.20, *p* = 0.38) and DFS (HR 1.25; 95% CI= 0.60–2.60, *p* = 0.55) were improved in the group adding consolidation chemotherapy compared with the standard CRT group, as well as with a decreased distant metastasis (*p* = 0.048) for early stage cervical cancer patients. Recently, an Asian Gynecologic Oncology Group study was carried out to determine whether only adding gemcitabine in the CRT phase without adjuvant chemotherapy could improve survival for advanced cervical cancer [19]. An interim analysis showed a slight improvement on the 3-year PFS (CRT 65.1% vs. CRT plus gemcitabine 71.0%, *p* = 0.71) and OS (CRT 74.1% vs. CRT plus gemcitabine 85.9 %, *p* = 0.89) in CRT plus gemcitabine arm. Additionally, the failure patterns were similar in both arms. This study further confirmed that adding consolidation chemotherapy may enhance the local control, decrease distant metastases and therefore improves patient survival.

The OS and DFS improvement in our study was lack of statistical significance, and only distant alone recurrence was decreased in the study group, while the other failure patterns were similar in both arms. This may due to a relative small number of patients enrolled with a relatively short follow-up time. Jelavić TB et al presented long-term outcomes of treatment with concomitant CRT followed by consolidation chemotherapy regimen, in which 15 out of 118 advanced cervical cancer patients developed distant recurrence alone. It achieved a distant disease-specific survival of 86.4% according to a median follow-up of 96 months [20]. The authors suggested that consolidation chemotherapy following concomitant CRT had a potential role in further improving control of the disease, especially the control of the distant metastasis.

In this study, pathologic type and pelvic nodes involvement were shown to be significant prognostic factors associated with OS according to multivariate analysis. This result was consistent with several previous studies analyzing prognostic factors in cervical cancer patients. Mabuchi et al reported that adenocarcinoma histology was significantly associated with the decreased disease-specific survival compared with squamous cell carcinoma histology in the intermediate- and high-risk cervical cancer (HR: 3.06 and 2.88, respectively, both *P* < 0.05) [21]. Yi-Jun Kim et al also demonstrated that lymph node metastasis was related with a higher distant metastasis [22].

As chemotherapy can cause toxicities, potential survival advantages must outweigh these disadvantages. Compliance of the experimental regimen in our study was acceptable with 90.1% of patients completed allocated consolidation chemotherapy. Toxicities in the consolidation group were not different from those found in the standard CRT group, though myelosuppression of Grade 3–4 was slightly more common (*p* = 0.053). Most of these adverse events were self-limiting or settled with medical management. Similarly, Lorvidhaya reported that hematological toxicities were not increased by consolidation chemotherapy after CRT and late side effects were similar between studied arms [23]. A retrospective matched-case comparison also found that toxicities in the consolidation group were not different from those found in the standard CRT group, though neutropenia of Grade 3 was slightly more common (*p* = 0.07) [11]. Future studies with novel chemotherapy combination could be designed in order to provide better clinical utility.

This trial had some limitations that should be carefully discussed. First, as we mentioned before, the sample size was relatively small to obtain a confirmative conclusion. Second, there were some possible bias because this was a nonblinded study design, despite the enrollment criteria and outcome measurements had been defined as objectively as possible. In conclusion, our study demonstrated a trend of improved OS and DFS for high-risk early stage cervical cancer patients treated with CRT plus consolidation chemotherapy. The paclitaxel/cisplatin combination was well tolerated overall and may play a potential role in further improving survival outcomes, especially the distant control of the disease. Future studies with large sample size and long follow up are needed to confirm these impressions.

## MATERIALS AND METHODS

### Eligibility criteria

The eligibility criteria were as follows: (1) women who were 18–75 years old underwent radical hysterectomy with diagnosis of invasive cervical cancer (non-small cell type); (2) with a postoperative pathological diagnosis of FIGO stage IA2 to IIB; (3) with positive pelvic nodes and/or positive surgical margin and/or positive parametrium. Additional requirements for eligibility were an Eastern Cooperative Oncology Group performance status 0–2; adequate function of major organs (including cardiac, hepatic and renal functions); hemoglobin level ≥ 10.0 g/dL, normal white blood cell and platelet count. All patients underwent chest radiographs and abdominopelvic computed tomography (CT) to exclude distant metastases. Patients with previous history of chemotherapy or radiation were excluded from this study.

This study was carried out according to ethical standards, national and international guidelines. It was approved by the institutional review board of our institution and undertaken in accordance with the Declaration of Helsinki. Written informed consent was obtained from each patient before treatment. A more comprehensive list of inclusion and exclusion criteria for this clinical trial is available on the ClinicalTrials.gov with an Identifier: NCT01755845.

### Treatment schemes and assessment

All patients underwent type C radical hysterectomy plus bilateral pelvic lymph node dissection. Paraaortic lymphadenectomy was not routinely performed. Pelvic lymph node dissection included removal of all the external iliac, internal iliac, common iliac, obturator, suprainguinal, and presacral lymph nodes. Patients were randomly assigned to arm A or arm B using a computerized number generator through the stratified block randomization method of the SAS package (SAS Institute Inc., Cary, North Carolina, USA) by a statistician with no clinical involvement in this trial. For radiotherapy, all patients were treated with external-beam radiotherapy (EBRT) at a daily fraction of 2.0 Gy for a total dose of 46–50 Gy. The treatment field was categorized as extended fields (para-aortic region plus whole pelvis) or whole pelvis on the basis of the level of LN metastasis. The superior border of the whole pelvis field was at the L4–L5 interspace. The inferior border was either at the lower border of the ischial tuberosity or the lower border of the obturator foramen depending on the extension of the vagina. The lateral portal anterior margin was plated at the pubic symphysis, and the posterior margin was at the S2–S3 interspace. Extended field radiotherapy was decided by radiation oncologists based on clinical necessary. When common iliac or para-aortic lymph nodes (PALN) were suspected to be involved from imaging studies, irradiation fields were extended to the abdominal para-aortic region. Pelvic EBRT was delivered mainly by a four-field box technique at 6 MV on a linear accelerator. One patient with positive surgical margin and two with close vaginal mucosal surgical margins were boosted with a brachytherapy dose of 30 Gy in 6 fractions.

For chemotherapy, patients randomized to arm B were administered paclitaxel 135 mg/m^2^ d1 and cisplatin 25 mg/m^2^ d1–3 intravenously every 4 weeks during radiation. After completion of CRT, consolidation chemotherapy consisted of paclitaxel 135 mg/m^2^ d1 and cisplatin 25 mg/m^2^ d1–3 repeating every 21 days for 2 courses was given in the absence of disease progression or unacceptable toxicity. Paclitaxel would be replaced by 5-FU if patients were allergic. If grade 4 toxicity occurred, the doses of chemotherapy were decreased by 10–15%. Patients randomly assigned to arm A received CRT alone, which was identical to that in arm B. Patients who developed recurrences within one year after therapy were treated with cisplatin/carboplatin and gemcitabine, while those who developed recurrences one year after received chemotherapy consisted of paclitaxel and cisplatin/carboplatin.

During treatment, all patients underwent weekly hematology and blood chemistry tests for safety and dose adjustment purposes. Treatment response was evaluated by WHO criteria with physical examinations. Follow-up was performed at 3-month intervals for the first 2 years, at 6-month intervals thereafter. Follow-up examinations consisted of physical examination, complete blood cell count, blood chemistries, tumor markers and abdomino-pelvic CT. Chest X-rays or CT scans, if indicated clinically, were performed every 6 months in the first 2 years then yearly to ensure recurrence data collection. A radionuclide bone scan and endoscopy were performed if clinically indicated. In the poststudy follow-up phase, all patients had evaluations for efficacy and safety end points approximately 30 days after completion of treatments and then approximately every 4 months for 12 months. Survival, disease recurrence, and post-therapy treatment were monitored every 6 months up to 48 months thereafter until death or study end.

The primary end point of the study was disease-free survival (DFS) at 3 years. The secondary efficacy measures were overall survival (OS) and toxicities. Safety was evaluated by recording clinical adverse events (AEs) using the National Cancer Institute Common Toxicity Criteria (version 3.0).

### Statistical methods

An interim safety analysis was performed in July 2015. Efficacy was analyzed in intention-to-treat population. DFS was calculated from the date of enrollment to the date of disease relapse (or death due to disease progression). OS was calculated from the date of enrollment to death for non-censored observations or censored at the date of last contact. The comparison of clinical characteristics between the two groups was based on a *t* test for continuous variables, and Pearson's chi-square test was used to evaluate the associations between categorical variables. Survival curves were estimated using Kaplan–Meier method and comparisons were made using the log-rank test. Cox regression was used to estimate hazard ratios (HRs). For multivariate analysis of prognostic factors, separate Cox proportional hazards regression models were utilized to estimate the relationship between each variables and OS or DFS. A probability (*P*) value of < 0.05 was considered statistically significant. All statistical analyses were performed using IBM SPSS version 22.0 (IBM, Armonk, NY, U.S.A.).

## References

[1] Jemal A, Bray F, Center MM, Ferlay J, Ward E, Forman D (2011). Global cancer statistics. CA Cancer J Clin.

[2] Xue F, Lin LL, Dehdashti F, Miller TR, Siegel BA, Grigsby PW (2006). F-18 fluorodeoxyglucose uptake in primary cervical cancer as an indicator of prognosis after radiation therapy. Gynecol Oncol.

[3] Schwarz JK, Siegel BA, Dehdashti F, Grigsby PW (2007). Association of posttherapy positron emission tomography with tumor response and survival in cervical carcinoma. Jama.

[4] Delgado G, Bundy B, Zaino R, Sevin BU, Creasman WT, Major F (1990). Prospective surgical-pathological study of disease-free interval in patients with stage IB squamous cell carcinoma of the cervix: a Gynecologic Oncology Group study. Gynecol Oncol.

[5] Rotman M, Sedlis A, Piedmonte MR, Bundy B, Lentz SS, Muderspach LI, Zaino RJ (2006). A phase III randomized trial of postoperative pelvic irradiation in Stage IB cervical carcinoma with poor prognostic features: follow-up of a gynecologic oncology group study. Int J Radiat Oncol Biol Phys.

[6] Sedlis A, Bundy BN, Rotman MZ, Lentz SS, Muderspach LI, Zaino RJ (1999). A randomized trial of pelvic radiation therapy versus no further therapy in selected patients with stage IB carcinoma of the cervix after radical hysterectomy and pelvic lymphadenectomy: A Gynecologic Oncology Group Study. Gynecol Oncol.

[7] Morris M, Eifel PJ, Lu J, Grigsby PW, Levenback C, Stevens RE, Rotman M, Gershenson DM, Mutch DG (1999). Pelvic radiation with concurrent chemotherapy compared with pelvic and para-aortic radiation for high-risk cervical cancer. N Engl J Med.

[8] Keys HM, Bundy BN, Stehman FB, Muderspach LI, Chafe WE, Suggs CL, Walker JL, Gersell D (1999). Cisplatin, radiation, and adjuvant hysterectomy compared with radiation and adjuvant hysterectomy for bulky stage IB cervical carcinoma. N Engl J Med.

[9] Rose PG (2002). Chemoradiotherapy for cervical cancer. Eur J Cancer.

[10] Duenas-Gonzalez A, Zarba JJ, Patel F, Alcedo JC, Beslija S, Casanova L, Pattaranutaporn P, Hameed S, Blair JM, Barraclough H, Orlando M (2011). Phase III, open-label, randomized study comparing concurrent gemcitabine plus cisplatin and radiation followed by adjuvant gemcitabine and cisplatin versus concurrent cisplatin and radiation in patients with stage IIB to IVA carcinoma of the cervix. J Clin Oncol.

[11] Choi CH, Lee YY, Kim MK, Kim TJ, Lee JW, Nam HR, Huh SJ, Lee JH, Bae DS, Kim BG (2011). A matched-case comparison to explore the role of consolidation chemotherapy after concurrent chemoradiotherapy in cervical cancer. Int J Radiat Oncol Biol Phys.

[12] Zhang MQ, Liu SP, Wang XE (2010). Concurrent chemoradiotherapy with paclitaxel and nedaplatin followed by consolidation chemotherapy in locally advanced squamous cell carcinoma of the uterine cervix: preliminary results of a phase II study. Int J Radiat Oncol Biol Phys.

[13] Vale C, Tierney JF, Stewart LA, Brady M, Dinshaw K, Jakobsen A, Parmar MKB, Thomas G, Trimble T, Alberts DS, Chen HW, Cikaric S, Eifel PJ (2008). Reducing Uncertainties About the Effects of Chemoradiotherapy for Cervical Cancer: A Systematic Review and Meta-Analysis of Individual Patient Data From 18 Randomized Trials. J Clin Oncol.

[14] Moore DH, Blessing JA, McQuellon RP, Thaler HT, Cella D, Benda J, Miller DS, Olt G, King S, Boggess JF, Rocereto TF (2004). Phase III study of cisplatin with or without paclitaxel in stage IVB, recurrent, or persistent squamous cell carcinoma of the cervix: A gynecologic oncology group study. J Clin Oncol.

[15] Rose PG (2006). Concurrent chemoradiation for locally advanced carcinoma of the cervix: where are we in 2006?. Ann Oncol.

[16] Rose PG, Bundy BN, Watkins EB, Thigpen JT, Deppe G, Maiman MA, Clarke-Pearson DL, Insalaco S (1999). Concurrent cisplatin-based radiotherapy and chemotherapy for locally advanced cervical cancer. N Engl J Med.

[17] Whitney CW, Sause W, Bundy BN, Malfetano JH, Hannigan EV, Fowler WC, Clarke-Pearson DL, Liao SY (1999). Randomized comparison of fluorouracil plus cisplatin versus hydroxyurea as an adjunct to radiation therapy in stage IIB-IVA carcinoma of the cervix with negative para-aortic lymph nodes: a Gynecologic Oncology Group and Southwest Oncology Group study. J Clin Oncol.

[18] Eifel PJ, Winter K, Morris M, Levenback C, Grigsby PW, Cooper J, Rotman M, Gershenson D, Mutch DG (2004). Pelvic irradiation with concurrent chemotherapy versus pelvic and para-aortic irradiation for high-risk cervical cancer: an update of radiation therapy oncology group trial (RTOG) 90–01. J Clin Oncol.

[19] Wang CC, Chou HH, Yang LY, Lin H, Liou WS, Tseng CW, Liu FY, Liou JD, Huang KG, Huang HJ, Huang EY, Chen CH, Chang TC (2015). A randomized trial comparing concurrent chemoradiotherapy with single-agent cisplatin versus cisplatin plus gemcitabine in patients with advanced cervical cancer: An Asian Gynecologic Oncology Group study. Gynecol Oncol.

[20] Jelavic TB, Mise BP, Strikic A, Ban M, Vrdoljak E (2015). Adjuvant Chemotherapy in Locally Advanced Cervical Cancer After Treatment with Concomitant Chemoradiotherapy—Room for Improvement?. Anticancer Res.

[21] Mabuchi S, Okazawa M, Matsuo K, Kawano M, Suzuki O, Miyatake T, Enomoto T, Kamiura S, Ogawa K, Kimura T (2012). Impact of histological subtype on survival of patients with surgically-treated stage IA2–IIB cervical cancer: adenocarcinoma versus squamous cell carcinoma. Gynecol Oncol.

[22] Kim YJ, Lee KJ, Park KR, Kim J, Jung W, Lee R, Kim SC, Moon HS, Ju W, Kim YH, Lee J (2015). Prognostic analysis of uterine cervical cancer treated with postoperative radiotherapy: importance of positive or close parametrial resection margin. Radiat Oncol J.

[23] Lorvidhaya V, Chitapanarux I, Sangruchi S, Lertsanguansinchai P, Kongthanarat Y, Tangkaratt S, Visetsiri E (2003). Concurrent mitomycin C, 5-fluorouracil, and radiotherapy in the treatment of locally advanced carcinoma of the cervix: A randomized trial. Int J Radiat Oncol.

